# Microbial Quality Assessment of Farmers′ Market Food Products in Central Virginia During the COVID‐19 Pandemic

**DOI:** 10.1155/ijfo/1807052

**Published:** 2026-05-06

**Authors:** Chyer Kim, Abeer Abujamous, Daria Clinkscales, Allissa Riley, Salina Parveen, Junglim Lee, Eunice Ndegwa, Theresa Nartea

**Affiliations:** ^1^ Agricultural Research Station, Virginia State University, Petersburg, Virginia, USA, tnau.ac.in; ^2^ Department of Nutrition and Food Sciences, University of Vermont, Burlington, Vermont, USA, uvm.edu; ^3^ Department of Agriculture, Food and Resource Sciences, University of Maryland Eastern Shore, Princess Anne, Maryland, USA, umes.edu; ^4^ Department of Human Ecology, Delaware State University, Dover, Delaware, USA, desu.edu; ^5^ Virginia Cooperative Extension, Virginia State University, Petersburg, Virginia, USA

**Keywords:** AMR, *Campylobacter*, *E. coli*, farmers′ market, food commodities, *Listeria*, *Salmonella*

## Abstract

Given the positive correlation between the rise in farmers′ markets and foodborne illness cases, it is crucial to assess the impact of personal protective equipment (PPE) use during the COVID‐19 pandemic on food microbial quality. This study is aimed at determining the prevalence and antimicrobial resistance (AMR) profiles of potential foodborne pathogens in a diverse range of food products obtained from farmers′ markets in Central Virginia during the COVID‐19 pandemic. A total of 740 food samples, comprising eight fresh produce types and four animal‐derived products, were randomly collected in duplicate from 15 registered farmers′ markets in Central Virginia (within a 50‐km radius of Virginia State University) between August 2020 and December 2021. The samples represented products from 76 farm operations. *Campylobacter*, *E. coli*, *Listeria*, and *Salmonella* were detected in the samples at 1.5%, 19.2%, 7.3%, and 0.8%. Compared with the previous findings between March–November 2017 (prepandemic), *Campylobacter* and *Listeria* prevalence decreased by about 3.6% and 5.5%, whereas *E. coli* and *Salmonella* increased by 1.7% and 0.3%. Resistance to ampicillin, streptomycin, nalidixic acid, and amoxicillin‐clavulanic acid was most common in 90.9% *Campylobacter*, 50.4% *E. coli*, 90.9% *Listeria*, and 66.7% *Salmonella* isolates, respectively. Overall, resistance to streptomycin was the most prevalent, seen in 48.8% of isolates, and about 7% exhibited multidrug resistance (MDR). None of the tested antimicrobials was universally effective against all bacterial species. Despite compliance with PPE protocols by vendors and consumers during the pandemic, foodborne pathogens with AMR continue to be detected in food commodities, highlighting the importance of ongoing research and education to address these issues and promote the safe development of farmers′ markets.

## 1. Introduction

The number of farmers′ markets in the United States has experienced a steady increase over the years, from 1755 in 1994 to 8711 in 2019 [1]. This increase was further amplified during the COVID‐19 pandemic, which saw record sales in these markets [2, 3]. Despite the ongoing pandemic in the year 2020, more than 18,160 farmers earned over $346 million by selling at farmers′ markets, whereas 34,860 farmers made $763 million through on‐farm and off‐farm stands [4]. Farmers′ markets are a crucial source of food products for many American communities, including Virginia. With 249 markets, Virginia ranks ninth among the Top 10 states for the number of farmers′ markets [5].

Consumers often perceive food products from farmers′ markets as being of higher quality due to their freshness and direct sourcing from farms [6]. Many support local farms for several reasons: the perceived freshness, health benefits, superior taste, and support for local economic development. Other reasons include confidence in the safety of local food, environmental benefits from reduced carbon emissions, fewer supply chain issues compared with grocery stores, and safer food during the COVID‐19 pandemic [2, 7, 8].

Despite these perceptions, continuing research is essential to understand the food safety and microbiological implications of products sold at farmers′ markets. These markets offer products processed by farmers on a relatively small scale, which often differ significantly from those produced by large‐scale operations. Small‐scale farming products are generally unregulated and often lack comprehensive agricultural and sanitation practices, increasing potential microbial risks [9].

Previous studies have highlighted the association between farmers′ markets and foodborne illness outbreaks. Therefore, there is a need for more research on the food safety risks associated with small farm production. Bennett et al. [10] found that many foodborne illnesses stemmed from vegetable row crops and fruits. Bellemare and Nguyen [11] reported a positive correlation between the number of farmers′ markets and reported cases of foodborne illness. Scheinberg et al. [12] revealed inadequate food safety behaviors among vendors at farmers′ markets, underscoring the need for improved food safety training. Historical data also indicate the presence of foodborne pathogens in these markets [13–22].

Additionally, antibiotic use in human and agricultural practices has led to widespread antimicrobial resistance (AMR), posing a significant public health threat, with the forecast of 10 million global deaths per year by 2050 [23], requiring action across all government sectors and society [24]. In the United States, over 3 million people acquire serious infections from AMR bacteria annually, with at least 48,000 deaths resulting directly from these infections [25].

Infections from AMR bacteria are no longer confined to only those associated with the medical community [26]. Ndegwa et al. [27] indicated that pastured goats, despite minimal exposure to antibiotics, are potential reservoirs of AMR *E. coli* and may contaminate the environment and food chain, spreading resistant genes to potentially pathogenic animal and human pathogens. In other studies, emerging AMR foodborne pathogens, including *Campylobacter*, *Listeria*, and *Salmonella*, associated with chicken meat [28], camel meat [29], fresh vegetables [30], and retail meats [31] have been reported. Furthermore, studies conducted at Virginia State University before the COVID pandemic revealed the presence of *Campylobacter*, *Escherichia coli*, *Listeria*, and *Salmonella* in environmental samples obtained from farm animals and wildlife [32], and food commodities obtained from farmers′ markets [9, 33]. These isolates exhibited high levels of AMR, with a significant presence of multidrug‐resistance (MDR).

At the beginning of the COVID‐19 pandemic, as a precaution to mitigate the spread of COVID, the World Health Organization [34] and the US Centers for Disease Control and Prevention [35] recommended personal hygienic practices and the rational use of personal protective equipment (PPE). Adhering to the recommendations, citizens started wearing PPE in addition to social distancing while shopping at food markets, including farmers′ markets. However, the effects of hygienic interventions, including hand sanitizer, PPE, and social distancing, on bacterial contamination levels and associated AMR in foods sold at farmers′ markets is unknown.

This study is therefore aimed at evaluating the prevalence and AMR of potential foodborne pathogens in food products randomly obtained from farmers′ markets in Central Virginia during the COVID‐19 pandemic. Additionally, the genomic diversity of most commonly found fecal contamination and hygienic condition indicating bacteria (*E. coli*, BAM 1998) and their strain relatedness associated with the products and market sources were evaluated to identify potential sources and niches. Furthermore, the microbial quality data of these food commodities, randomly procured from the same geographical locations before and during the pandemic, were compared with determine if the mandated use of PPE and social distancing during the pandemic influenced the microbial quality of food products from farmers′ markets.

## 2. Materials and Methods

### 2.1. Samples Used

Food commodities were randomly procured from local farmers′ markets located in Central Virginia within a radius of 50 km From Virginia State University (Petersburg, Virginia). Food commodities representing two categories—fresh produce (arugula, green beans, green onions, green peppers, kale, lettuce, squash, and tomatoes) and animal‐origin products (chicken, eggs, ground beef, and pork sausage)—were procured for analysis. All purchases were made in duplicate between August 2020 and December 2021. Purchased samples were transported to our laboratory in insulated containers packed with ice. All products were kept in the refrigerator (4^°^C ± 2^°^C) and used for microbial testing within 2 days of arrival.

### 2.2. Microbial Testing

Following the procedure described by Kim et al. [9, 33, 36, 37], samples were prepared for microbial analysis. In brief, blendable samples (arugula, lettuce, ground beef, and pork sausage) were composited, and 25 g portions were diluted 1:10 in sterile 0.1% peptone water (PW) and homogenized for 2 min at 260 rpm using a laboratory blender (Model 400 Circulator, Seward Ltd., United Kingdom). Nonblendable samples such as green beans, green onions, green peppers, kale, squash, tomatoes, chicken, and eggs; each whole sample was aseptically transferred into a stomacher bag containing an equal weight of sterile PW. The samples were then agitated and vigorously rubbed by a gloved hand for 2 min to detach microorganisms [38].

Serial dilutions of sample homogenates or wash fluids were plated on standard method agar (SMA; BD, Sparks, MD) for enumeration of aerobic mesophiles following incubation at 36°C for 48 h. Detection limits were 2 log CFU/g for blendable samples and 1.3 log CFU/mL for nonblendable samples. Total coliforms and *E. coli* were quantified using a three‐tube most‐probable‐number (MPN) assay initiated in lauryl sulfate tryptose (LST) broth. Presumptive positive tubes were confirmed in brilliant green bile broth for total coliforms and in EC‐mug broth for *E. coli* following incubation at 36°C and 45.5°C, respectively, with fluorescence under UV light (365 nm) indicating *E. coli* [9, 33]. All positive EC tubes were streaked on eosin–methylene blue agar, and purple colonies (with or without a green metallic sheen) were evaluated using API 20E test kits (bioMérieux, Hazelwood, MO) for *E. coli* confirmation. From the three highest EC‐MPN dilution tubes, a single confirmed isolate was randomly chosen from each positive tube and retained for subsequent AMR analysis.

The occurrence of *Campylobacter*, *Listeria*, and *Salmonella* was assessed due to their relevance to severe foodborne illness [39]. For *Campylobacter* detection, 25 g of blendable samples or entire nonblendable samples were enriched in modified Bolton broth (CM0983, Oxoid Ltd., Basingstoke, Hampshire, England) supplemented with laked horse blood (Biological Laboratories, Pipersville, Pennsylvania) and selective supplement (SR0183E, Oxoid Ltd.), followed by incubation at 42°C for 48 h. Enrichments were streaked onto selective *Campylobacter* agar supplemented with cefoperazone and amphotericin B (SR0155, Oxoid Ltd.) and incubated microaerobically at 42°C for 48 h. Presumptive *Campylobacter* isolates were identified based on characteristic colony appearance and gram‐negative, curved cell morphology observed by light microscopy [40].

For further confirmation of *Campylobacter* [41], genomic DNA from presumptive *Campylobacter* isolates was obtained using a heat‐lysis procedure. Enrichment cultures were pelleted by centrifugation at 11,180 × g for 4 min, washed twice with molecular‐grade water, and resuspended in 300 *μ*L of water. Suspensions were heated at 100°C for 20 min and centrifuged at high speed (23,831 × g) for 4 min, after which the supernatant containing DNA was recovered. DNA concentration was determined using a NanoDrop 2000C spectrophotometer (Thermo Scientific, Waltham, Massachusetts, United States), and extracts were stored at −80°C prior to PCR analysis.

Presumptive *Campylobacter* isolates were confirmed by PCR targeting the conserved 23sRNA gene. Species‐level identification of *C. jejuni* and *C. fetus* was performed using SYBR Green–based real‐time PCR assays (A25741, SYBR Green PCR Master Mix‐Life Technologies, Massachusetts, United States) with species‐specific primers. Detection of *C. jejuni* utilized forward (5 ^′^‐TATACCGGTAAGGAGTGCTGGAG‐3 ^′^) and reverse (5 ^′^‐ATCAATTAACCTTCGAGCACCG‐3 ^′^) primers, amplifying an approximately 650‐bp fragment. Identification of *C. fetus* was performed using the primer pair 5 ^′^‐GCAAATATAAATGTAAGCGGAGAG‐3 ^′^ (forward) and 5 ^′^‐TGCAGCGGCCCCACCTAT‐3 ^′^ (reverse).

Conventional PCR was conducted using Amplitaq 360 Gold master mix at an annealing temperature of 60°C, with appropriate positive and negative controls included. Amplification products were resolved on 1.5% agarose gels and visualized under UV illumination (Life Technologies Corp., Neve Yamin, Israel).

For *Listeria* detection, each sample (25 g for blendable samples or the whole sample for nonblendable samples) was prepared in the appropriate amount of sterile University of Vermont Medium (UVM) broth as previously described and enriched at 30°C for 48 h. A loopful of the enrichment broth was then surface streaked onto Oxford *Listeria* agar for isolation. Presumptive *Listeria* colonies (brown colonies with black zones) were identified to species level using the API Listeria kit.

For *Salmonella* detection, each sample (25 g for blendable samples or the whole sample for nonblendable samples) was prepared in the appropriate amount of sterile buffered peptone water (BPW) as described above, pre‐enriched at 36°C for 24 h, and enriched in Rappaport‐Vassiliadis (RV) broth at 42°C for 18 h. RV broth cultures were then surface streaked onto xylose lysine desoxycholate agar for isolation. Presumptive *Salmonella* colonies (those with black centers) were confirmed using the API 20E test kits.

Confirmed *Campylobacter*, *E. coli*, *Listeria*, and *Salmonella* isolates were preserved in *Brucella* broth supplemented with 20% glycerol and maintained at −80°C until AMR characterization.

### 2.3. AMR

Following the procedure outlined by Kim et al. [9], antimicrobial susceptibility tests were conducted using the Kirby–Bauer disk diffusion method [42]. In brief, the confirmed isolates of *Campylobacter*, *E. coli*, *Listeria*, and *Salmonella* were tested for susceptibility to 12 antimicrobial agents representing nine different categories (Table 1). The antimicrobial agents obtained from Oxoid and tested were: ampicillin (AMP), amoxicillin‐clavulanic acid (AMC), meropenem (MEM), amikacin (AMK), gentamicin (GEN), streptomycin (STR), tobramycin (TOB), tetracycline (TCY), ciprofloxacin (CIP), nalidixic acid (NAL), chloramphenicol (CHL), and trimethoprim‐sulfamethoxazole (SXT). The results of the antimicrobial susceptibility were classified as “resistant,” “intermediate,” or “susceptible” according to the criteria established by the National Committee for Clinical Laboratory Standards (NCCLS) [42]. Additionally, bacteria classified as either resistant or intermediate were defined as “non‐susceptible,” and those exhibiting resistance to at least one antimicrobial agent in three or more categories were defined as MDR [43, 44]. *E. coli* ATCC 25922 was used as a control strain for the performance of antimicrobials in this study.

**Table 1:** A list of antimicrobials and interpretive criteria used in this study (CLSI 2015).

Antimicrobial category	Antimicrobial agent and its abbreviation	Concentration (*μ*g/disk)	Zone diameter (mm)		
			S	I	R
Penicillins	Ampicillin (AMP)	10	> 17	14–16	< 13
*β*‐lactamase inhibitor combinations	Amoxicillin‐clavulanic acid (AMC)	30	> 18	14–17	< 13
Carbapenems	Meropenem (MEM)	10	> 23	20–22	< 19
Aminoglycosides	Amikacin (AMK)	30	> 17	15–16	< 14
	Gentamicin (GEN)	10	> 15	13–14	< 12
	Streptomycin (STR)	10	> 15	12–14	< 11
	Tobramycin (TOB)	10	> 15	13–14	< 12
Tetracyclines	Tetracycline (TCY)	30	> 15	12–14	< 11
Fluoroquinolones	Ciprofloxacin (CIP)	5	> 21	16–20	< 15
Quinolones	Nalidixic acid (NAL)	30	> 19	14‐18	< 13
Phenicols	Chloramphenicol (CHL)	30	> 18	13‐17	< 12
Folate pathway inhibitors	Trimethoprim‐sulfamethoxazole (SXT)	25	>16	11–15	< 10

*Note:* Interpretive criteria: S, susceptible; I, intermediate; and R, resistant to antimicrobial agents tested.

#### 2.3.1. *Campylobacter*

*Campylobacter* cultures propagated in modified Bolton broth at 42°C for 48 h were standardized to approximately 8 log CFU/mL, and a 100 *μ*L aliquot was spread onto blood agar plates. After allowing the inoculum to absorb for 10 min, antimicrobial disks were placed using a multidisk dispenser. Plates were incubated microaerobically at 42°C for 24 h, after which inhibition zone diameters were measured in millimeters using calipers and recorded for each isolate.

#### 2.3.2. *E. coli*, *Listeria*, and *Salmonella*

Confirmed *E. coli*, *Listeria*, and *Salmonella* isolates were inoculated into Mueller–Hinton broth (MHB) and incubated at 36°C for 24 h, followed by subculture under the same conditions to ensure culture viability prior to antimicrobial susceptibility testing. *E. coli* ATCC 25922 was prepared in parallel as a quality‐control strain. Cultures were standardized to approximately 8 log CFU/mL, and 100 *μ*L aliquots were evenly spread onto Mueller–Hinton agar (MHA) plates. After a 10‐min absorption period, antimicrobial disks were applied using an automated multidisk dispenser. Plates were incubated at 36°C for 24 h, and inhibition zone diameters were measured in millimeters with calipers and documented for each isolate.

### 2.4. Data Analysis

Mean microbial counts (aerobic mesophiles, coliforms, and *E. coli*) were calculated from duplicate samples following log transformation. Statistical comparisons were conducted using analysis of variance with Duncan′s multiple range test to determine significant differences (*p* < 0.05) (SAS Institute, Cary, North Carolina, United States). Values below the detection or quantification limit were assigned the corresponding limit for analysis. Associations among the presence of *Campylobacter*, *E. coli*, *Listeria*, and *Salmonella* were evaluated by correlation analysis using binary coding (absent = 0; present = 1).

## 3. Results and Discussion

**Figure 1:**
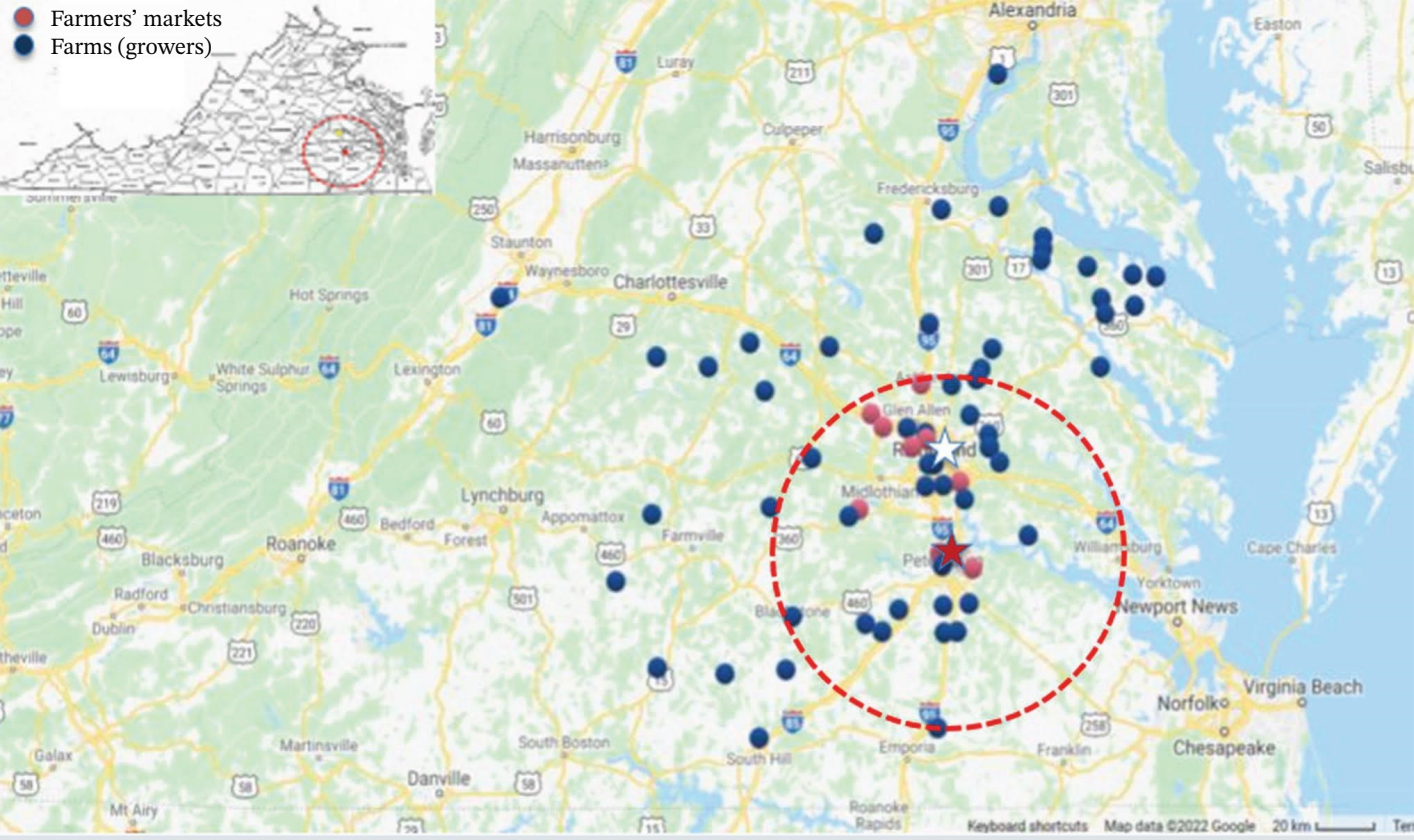
Map of Virginia (Source: https://legacy.lib.utexas.edu/maps/states/virginia.gif) showing locations of farmers′ markets [red‐dashed area, within a radius of 50 km from Virginia State University] where food product samples were obtained. Location of representative farmers′ markets and farms (growers) are indicated in red and blue dots, respectively. Richmond (in white star), Virginia′s capital city and Virginia State University (in red star) located in the city of Petersburg are indicated as location references. Map was generated using a Maptive software.

Results of the levels of aerobic mesophile, coliform, and *E. coli* counts in the 740 samples analyzed are shown in Table 1. A total of 740 food commodities were randomly procured from 15 registered local farmers′ markets located in the studied area. The general areas of studied farmers′ markets are depicted in Figure 1. The procured food commodities included 12 different types, consisting of eight types of fresh produce and four types of animal‐origin products, produced by 76 farm operations. The food commodities procured are shown in Table 2.

**Table 2:** The level of bacterial populations recovered from selected food products procured from farmers′ markets in Virginia between August 2020 and December 2021.

Sample type (*n* ^a^)	Sample ID (*n*)	Microbial population (Log CFU/g or Log MPN/g)/number of samples						
		Aerobic mesophiles			Coliform		*E. coli*	
		Mean^b^	Range	Occurrence (%)^c^	Mean	Range	Mean	Range
Produce (476)	Arugula (60)	6.73 ± 0.70 a	5.07–8.47	0.0, 63.3, 36.7	2.64 ± 1.38 bc	< 0.48– >5.04	0.72 ± 0.56 bc	< 0.48–2.88
	Green bean (62)	6.75 ± 0.54 a	5.65–7.81	0.0, 62.9, 37.1	2.44 ± 1.30 c	< 0.48–5.04	0.70 ± 0.48 bc	< 0.48–3.04
	Green onion (56)	6.74 ± 0.60 a	5.52–8.22	0.0, 67.9, 32.1	2.95 ± 1.58 ab	< 0.48– >5.04	0.65 ± 0.65 c	< 0.48–4.66
	Green pepper (62)	5.67 ± 0.79 c	4.34–7.18	24.2, 71.0, 4.8	2.36 ± 1.54 c	< 0.48–> 5.04	1.00 ± 1.16 b	< 0.48–5.04
	Kale (46)	6.16 ± 0.91 b	3.86–8.21	8.7, 71.7, 19.6	2.38 ± 1.50 c	< 0.48– >5.04	0.81 ± 0.80 bc	< 0.48–4.04
	Lettuce (46)	6.67 ± 0.73 a	4.71–7.88	2.2, 65.2, 32.6	3.13 ± 1.50 ab	0.56– >5.04	0.74 ± 0.51 bc	< 0.48–2.38
	Squash (66)	6.32 ± 0.51 b	4.28–7.19	1.5, 95.5, 3.0	3.24 ± 1.41 a	< 0.48–5.04	1.31 ± 1.18 a	< 0.48–5.04
	Tomato (78)	5.27 ± 0.71 d	3.00–6.68	28.2, 71.8, 0.0	1.77 ± 1.36 d	< 0.48–5.04	0.73 ± 0.58 bc	< 0.48–3.32
Animal‐ origin (264)	Chicken (40)	3.59 ± 1.49 c	1.60–6.90	80.0, 20.0, 0.0	1.81 ± 1.06 a	< 0.48– > 5.04	1.38 ± 1.01 a	< 0.48–4.04
	Egg (88)	3.08 ± 1.39 d	1.40–6.76	89.8, 10.2, 0.0	0.72 ± 0.80 b	< 0.48–5.04	0.65 ± 0.65 b	< 0.48–4.04
	Ground beef (50)	6.79 ± 0.77 a	4.86–7.90	6.0, 44.0, 50.0	2.04 ± 1.27 a	< 0.48–5.04	0.75 ± 0.77 b	< 0.48–4.18
	Pork sausage (86)	5.91 ± 1.31 b	3.18–8.64	20.9, 60.5, 18.6	1.65 ± 1.28 a	< 0.48–4.66	0.85 ± 0.70 b	< 0.48–3.66

*Note:* A total of 740 food commodities consisted of 476 produce and 264 animal origin items were randomly procured from 15 registered local farmers′ markets located in Central Virginia within a radius of 50 km from Virginia State University (Petersburg, Virginia). Products procured represented 12 different types of food commodities produced by 76 farm operations.

^a^Number of samples tested.

^b^Values are mean ± standard error of duplicate samples; means followed by the same lower‐case letters in the same column of each sample type are not significantly different (*p >0.05*).

^c^Values are the percentages of samples with aerobic mesophile counts within the range ≤ 105 CFU/g, 10^5^ to 10^7^ CFU/g, and ≥ 107 CFU/g, respectively.

**Table 3:** Prevalence of bacteria (*Campylobacter, E. coli*, *Listeria,* and *Salmonella*) in the samples analyzed.

Sample type (*n* ^a^)	Sample ID (*n*)	Occurrence of bacteria				
		*Campylobacter*	*E. coli*	*Listeria* spp.	*L. monocytogenes*	*Salmonella*
Produce (476)	Arugula (60)	ND^b^	8 (13.3%)	3 (5.0%)	ND	ND
	Green bean (62)	ND	12 (19.4%)	6 (9.7%)	1 (1.6%)	ND
	Green onion (56)	1 (1.8%)	1 (1.8%)	1 (1.8%)	1 (1.8%)	ND
	Green pepper (62)	ND	4 (6.5%)	ND	ND	ND
	Kale (46)	ND	10 (21.7%)	1 (2.2%)	ND	2 (4.3%)
	Lettuce (46)	3 (6.5%)	12 (26.1%)	3 (6.5%)	2 (4.3%)	1 (2.2%)
	Squash (66)	5 (7.6%)	14 (21.2%)	4 (6.1%)	2 (3.0%)	ND
	Tomato (78)	2 (2.6%)	5 (6.4%)	ND	ND	ND
	**Total (476)**	**11 (2.3%)**	**66 (13.9%)**	**18 (3.8%)**	**6 (1.3%)**	**3 (0.6%)**
Animal‐origin (264)	Chicken (40)	ND	31 (77.5%)	4 (10.0%)	1 (2.5%)	1 (2.5%)
	Egg (88)	ND	7 (8.0%)	ND	ND	ND
	Ground beef (50)	ND	6 (12.0%)	12 (24.0%)	5 (10.0%)	2 (4.0%)
	Pork sausage (86)	ND	32 (37.2%)	20 (23.3%)	8 (9.3%)	ND
	**Total (264)**	**0 (0.0%)**	**76 (28.8%)**	**36 (13.6%)**	**14 (5.3%)**	**3 (1.1%)**
	**Overall (740)**	**11 (1.5%)**	**142 (19.2%)**	**54 (7.3%)**	**20 (2.7%)**	**6 (0.8%)**

*Note:* The bolded values represent the total prevalence and overall summary of bacteria isolated from tested samples across the studied region during the sampling period. These values are highlighted to emphasize the aggregate data for bacteria recovered from produce and animal‑origin sources.

^a^Number of samples tested.

^b^ND: not detected.

**Table 4:** Antimicrobial resistance prevalence of 11 *Campylobacter*, 316 *E. coli*, 54 *Listeria*, and 6 *Salmonella* isolates in selected food samples procured from farmers′ markets in Central Virginia between August 2020 and December 2021.

Sample type	Bacteria (*n* ^a^)	Nature of AMR^b^	Prevalence (%) of resistance or nonsusceptibility to each quantity of antimicrobial agents^c^									
			1	2	3	4	5	6	7	8	9	MDR (≥ 3)^d^
Produce	*Campylobacter* spp. (11)	R	0.0	18.2	9.1	45.5	27.3	0.0	0.0	0.0	0.0	81.8
		*R* + *I*	0.0	0.0	0.0	18.2	45.5	27.3	0.0	9.1	0.0	NA^e^
	*E. coli* spp. (138)	R	18.8	5.8	0.7	0.0	0.0	0.0	0.0	0.0	0.0	1.4
		*R* + *I*	26.1	18.8	11.6	9.4	3.6	0.0	0.7	0.0	0.0	NA
	*Listeria* spp. (18)	R	50.0	5.6	5.6	0.0	5.6	22.2	5.6	5.6	0.0	44.4
		*R* + *I*	27.8	16.7	5.6	11.1	0.0	5.6	11.1	22.2	0.0	NA
	*Salmonella* spp. (3)	R	66.7	0.0	0.0	0.0	33.3	0.0	0.0	0.0	0.0	33.3
		*R* + *I*	0.0	33.3	33.3	0.0	0.0	0.0	33.3	0.0	0.0	NA
Animal‐origin	*E. coli* spp. (178)	R	6.2	8.4	2.2	0.0	0.0	0.0	0.0	0.0	0.0	2.8
		*R* + *I*	17.4	23.6	15.7	12.4	6.7	2.8	1.7	0.0	0.6	NA
	*Listeria* spp. (36)	R	72.2	11.1	0.0	0.0	0.0	8.3	2.8	0.0	0.0	11.1
		*R* + *I*	30.6	38.9	13.9	0.0	2.8	2.8	8.3	0.0	0.0	NA
	*Salmonella* spp. (3)	R	0.0	33.3	33.3	0.0	0.0	0.0	0.0	0.0	0.0	33.3
		*R* + *I*	33.3	0.0	0.0	0.0	0.0	66.7	0.0	0.0	0.0	NA
Combined	*Campylobacter* spp. (11)	R	0.0	18.2	9.1	45.5	27.3	0.0	0.0	0.0	0.0	81.8
		*R* + *I*	0.0	0.0	0.0	18.2	45.5	27.3	0.0	9.1	0.0	NA^e^
	*E. coli* spp. (316)	R	11.7	7.3	1.6	0.0	0.0	0.0	0.0	0.0	0.0	2.2
		*R* + *I*	21.2	21.5	13.9	11.1	5.4	1.6	1.3	0.0	0.3	NA
	*Listeria* spp. (54)	R	64.8	9.3	1.9	0.0	1.9	13.0	3.7	1.9	0.0	22.2
		*R* + *I*	29.6	31.5	11.1	3.7	1.9	3.7	9.3	7.4	0.0	NA
	*Salmonella* spp. (6)	R	33.3	16.7	16.7	0.0	16.7	0.0	0.0	0.0	0.0	33.3
		*R* + *I*	16.7	16.7	16.7	0.0	33.3	0.0	16.7	0.0	0.0	NA

*Note:* Susceptibility categorization was carried out in accordance with interpretive criteria provided by the National Committee of Clinical Laboratory Standards recommendations (CLSI 2015).

^a^Number of isolates tested.

^b^Antimicrobial resistance (AMR); R, resistant; I, intermediate; R + I, nonsusceptible to antimicrobial agents tested.

^c^Prevalence (%) was presented in resistance and nonsusceptibility of isolates to the total number of antimicrobial agents tested [i.e., an isolate exhibiting resistant and intermediate, respectively, to two and four antimicrobial agents was presented under 2 of resistance and 6 of nonsusceptibility (R + I).].

^d^Multidrug resistance.

^e^Not applicable.

### 3.1. Aerobic Mesophile Counts

Overall, there was considerable variability in aerobic mesophile counts across different food commodities sold at farmers′ markets in central Virginia. Among fresh produce, the mean aerobic mesophile counts for arugula (6.73 ± 0.70 log CFU/g), green bean (6.75 ± 0.54 log CFU/g), green onion (6.74 ± 0.60 log CFU/g), and lettuce (6.67 ± 0.73 log CFU/g) were significantly higher (*p* < 0.05) compared with those for green pepper (5.67 ± 0.79 log CFU/g), kale (6.16 ± 0.91 log CFU/g), squash (6.32 ± 0.51 log CFU/g), and tomato (5.27 ± 0.71 log CFU/g), with the highest counts observed in green beans and the lowest in tomatoes.

The International Commission on Microbiological Specifications for Foods [45] does not set a specific limit for aerobic mesophile counts in raw produce with unknown production and processing histories. However, to provide a better understanding of the microbiological quality of the samples acquired for this study, the aerobic mesophile counts are categorized and presented in Table 1 as follows: ≤ 10^5^ CFU/g, 10^5^ to 10^7^ CFU/g, and ≥ 107 CFU/g. The majority (63.3%–95.5%) of produce samples exhibited aerobic mesophile counts ranging between 5 and 7 log CFU/g. A small proportion (0.0%–4.8%) of tomato, squash, and green pepper samples had counts above 7 log CFU/g, whereas a comparatively higher proportion (19.6%–37.1%) of other produce samples (kale, green onion, lettuce, green beans, and arugula) exceeded this threshold. These results align with our previous findings [36] and suggest that commodities grown closer to the ground may have a greater likelihood of exposure to contamination sources, resulting in higher mesophilic bacterial counts. In contrast, tomatoes, squash, and peppers, which develop further from the ground surface, generally exhibit lower microbial loads.

For animal‐origin samples, the mean aerobic mesophile counts were significantly higher (*p* < 0.05) in ground beef (6.79 ± 0.77 log CFU/g), followed by pork sausage (5.91 ± 1.31 log CFU/g), chicken (3.59 ± 1.49 log CFU/g), and egg (3.08 ± 1.39 log CFU/g). Although there was limited availability of the same commodities at different farmers′ markets in the studied area, based on the limits established by the ICMSF, aerobic mesophile counts in the majority of samples were in the acceptable range (either ≤ 5 log CFU/g or 5–7 log CFU/g), but 50% and 18.6% of ground beef and pork sausage samples, respectively, had the unacceptable levels (> 7 log CFU/g). The aerobic mesophile counts in four out of 86 pork sausage samples (4.7%) were even greater than 8.0 log CFU/g. Consistent with our findings, a study conducted in Virginia [37] also reported high levels of aerobic mesophile counts (> 7 log CFU/g) in 37.5% of pork sausage samples from food desert markets, including supermarkets and corner stores in the same region.

It is noted that although among fresh produce samples, kale exhibited the broadest range of aerobic mesophile counts (3.86–8.21 log CFU/g), the overall range observed in animal‐origin samples was generally wider than those of fresh produce. Specifically, the range of the counts for animal‐origin samples were 1.60–6.90 log CFU/g for chicken, 1.40–6.76 log CFU/g for egg, 4.86–7.90 log CFU/g for ground beef, and 3.18–8.64 log CFU/g for pork sausage. Additionally, despite having high bacterial counts (> 8.0 log CFU/g), samples such as arugula, green onion, kale, and pork sausage showed no sensory signs of defect or spoilage.

### 3.2. Coliform Counts

Overall, 23.2% (172 out of 740) of the samples tested positive for coliforms, including 13.0% (62 out of 476) of fresh produce samples and 41.7% (110 out of 264) of animal‐origin samples. Among the produce samples, coliform levels in squash (3.24 ± 1.41 log MPN/g), lettuce (3.13 ± 1.50 log MPN/g), and green onion (2.95 ± 1.58 log MPN/g) were significantly higher (*p* < 0.05) than those in green bean (2.44 ± 1.30 log MPN/g), green pepper (2.36 ± 1.54 log MPN/g), kale (2.38 ± 1.50 log MPN/g), and tomato (1.77 ± 1.36 log MPN/g), with the highest counts observed in squash and the lowest in tomatoes (Table 1). Notably, at least one sample from the majority of the produce types [5 (arugula, green onion, green pepper, kale, and lettuce) out of 8 produce types] had coliform counts exceeding 5.04 log MPN/g. All commodities exhibited wide ranges of coliform counts, spanning from less than 0.5 to more than 5.0 log MPN/g. These results were somewhat similar to other investigations, where most of the fresh produce showed coliform counts from 2 to 6 log CFU/g [20, 36, 46, 47].

The mean coliform counts for lettuce (3.13 ± 1.50 log MPN/g) and kale (2.38 ± 1.50 log MPN/g) obtained in our study were considerably higher than 1.68 log MPN/g reported by Scheinberg et al. [48] in Pennsylvania, where 152 samples of leafy vegetables, including lettuce and kale obtained from farmers′ markets, were analyzed. Similarly, although Roth et al. [20] detected a mean coliform count of 2.3 log MPN/g in leafy greens sold at farmers′ markets in Florida, Pan et al. [49] reported the presence of fecal coliforms in 100% of 242 vegetable samples from six farmers′ markets in northern California. One possible explanation for the coliform counts is that leafy vegetables with large surface areas and folds are particularly prone to bacterial contamination and adhesion [50]. The open structure of the leaves allows contact with soil and irrigation water, leading to dirt accumulation in the folds [51]. Aycicek et al. [50] indicated that this risk is heightened when untreated manure is applied as fertilizer in the fields. Additionally, high mean coliform counts are concerning for food safety, as the presence of coliforms may indicate harmful, disease‐causing microorganisms [52, 53]. Furthermore, although only a small number (2 out of 78, 2.6%) of tomatoes from farmers′ markets had coliform counts reaching 5.04 log MPN/g, it is concerning given that these commodities may be consumed as ready‐to‐eat foods.

For animal‐origin samples, coliform levels in ground beef (2.04 ± 1.27 log MPN/g), chicken (1.81 ± 1.06 log MPN/g), and pork sausage (1.65 ± 1.28 log MPN/g) were significantly (*p* < 0.05) higher than that in eggs (0.72 ± 0.80 log MPN/g), with the highest counts observed in ground beef and the lowest in eggs. Although only one egg tested had coliform counts reaching 5.04 log MPN/g, this finding is concerning. Coliform bacteria may originate from environmental sources and do not necessarily reflect fecal contamination. However, elevated coliform counts have been used as indicators of general hygiene status during food handling and postharvest practices [54, 55]. In some contexts, higher coliform levels have been associated with the potential co‐occurrence of other microbial hazards [52, 53], suggesting a possible risk of cross‐contamination among commodities in farmers′ market settings.

### 3.3. *E. coli* Counts

Among the fresh produce samples, squash exhibited the highest average *E. coli* counts (1.31 ± 1.18 log MPN/g), significantly higher (*p* < 0.05) than other samples (Table 1). Notably, at least one produce sample had *E. coli* counts exceeding 2.38 log MPN/g, with green pepper and squash showing the broadest range (< 0.48–5.04 log MPN/g). In samples of animal‐origin, chicken had the highest average *E. coli* counts (1.38 ± 1.01 log MPN/g), significantly greater (*p* < 0.05) than other animal‐origin samples. Additionally, at least one sample from animal‐origin samples had *E. coli* counts exceeding 3.66 log MPN/g. The mean *E. coli* counts in onion (0.65 ± 0.65 log MPN/g) and in eggs (0.65 ± 0.65 log MPN/g) were the lowest among the fresh produce and animal‐origin samples, respectively.

### 3.4. Bacterial Prevalence

The prevalence of bacteria in the samples analyzed in this study is presented in Table 3. Among the 476 fresh produce samples tested, *Campylobacter* spp., *E. coli*, *Listeria* spp., and *Salmonella* spp. were detected in 11 (2.3%), 66 (13.9%), 18 (3.8%), and 3 (0.6%), respectively. *Listeria monocytogenes* was found in approximately 1.3% of the samples. *Campylobacter*, which is commonly linked to outbreaks associated with poultry, dairy products, and seafood [56], was detected at a rate of 1.8% (1/56) in green onions, 6.5% (3/46) in lettuce, 7.6% (5/66) in squash, and 2.6% (2/78) in tomatoes. Among all *Campylobacter* detections, 45.5% (5/11) were identified in squash samples.

All types of fresh produce tested in this study had at least one instance of *E. coli*, with the highest detection rate in lettuce (26.1%, or 12/46) and the lowest in green onions (1.8%, or 1/56). However, the overall prevalence (13.9%) of *E. coli* obtained in our study was considerably lower than a study conducted by Pan et al. [49], which detected *E. coli* in 20% of 242 commodities including basil, beans, squash, okra, and cilantro obtained from farmers′ markets in Northern California. Additionally, a survey [48] in Pennsylvania, where a total of 152 samples of leafy vegetables obtained from farmers′ markets were analyzed, detected the prevalence of *E. coli* in 25% of samples. However, prevalence obtained in our study was comparable with the prevalence found in another extensive study [57] that surveyed more than 2000 produce samples from 63 farms in Minnesota and Wisconsin, including organic, semiorganic and conventional farms, which detected *E. coli* in 8% of samples. In similar farmers’ market studies, the prevalence of *E. coli* was found in 2.3% of 301 leafy greens and spinach [20], 27% of 133 fresh herb samples [58], and 7.7% of 600 fresh produce including green onion, lettuce, and spinach [59].

Regardless, the presence of *E. coli* (6.4% of 78 samples) in tomatoes detected in the current study is concerning. Since tomatoes are often consumed as ready‐to‐eat foods without any further processing of “kill step”, the presence of *E. coli* suggests possible fecal contamination during harvesting and/or handling. This finding should be taken seriously, as potential pathogens such as *E. coli* O157 can originate from the same contamination sources [52, 60–62].

Among the 18 (3.8%) of the 476 fresh produce samples that tested positive for *Listeria* spp., *L. floribensis*, *L. monocytogenes*, *L. inoculate*, and *L. welshimeri* were isolated from 2 (11.1%), 6 (33.3%), 4 (22.2%), and 6 (33.3%) samples, respectively. Positive samples include arugula, green bean, green onion, kale, lettuce, and squash. The most prevalent *Listeria* species in the samples were *L. monocytogenes* and *L. welshimeri*, each accounting for 33.3%. Similar to our findings, a study [36] conducted in Virginia, United States also reported the detection of *Listeria* spp. in kale, turnip, and bell pepper, with 3.3% of the 122 fresh produce samples obtained from food markets in food desert areas. In a study on the microbiological quality of 301 fresh produce samples from nine farmers′ markets in North and Central Florida, Roth et al. [20] detected *L. monocytogenes* in 3.9% and 2.6% of spinach and leafy greens, respectively. Although prevalence of *L. monocytogenes* was 2.1% and 0% for spinach and lettuce from Pennsylvania farmers′ markets, respectively [48], Thunberg et al. [63] detected a variety of *Listeria* spp. on produce samples obtained from farmers′ markets in Washington, D.C. Roth et al. [20] indicated that although *Listeria* spp. on fresh produce is not uncommon as it is widespread in nature including soil, the detection of *L. monocytogenes* in produce from farmers′ markets is particularly concerning for high risk groups such as the elderly, pregnant women, children, and individuals with compromised immune systems.

In assessing *Salmonella* presence, positive samples were identified in kale and lettuce. Our finding of *Salmonella* detected in 0.6% of 476 samples is comparable with a study by Levy et al. [58], which found *Salmonella* in 0.8% of 133 fresh herb samples from 13 farmers′ markets in Los Angeles, California and Seattle, Washington. Conversely, a prevalence study of 261 leafy green commodities from California′s central coast found no detectable *Salmonella* [64], whereas Pan et al. [49] reported *Salmonella* in 6.6% of 242 fresh produce samples obtained from farmers′ markets in Northern California.

Meanwhile, it is noted that two samples of kale and one sample of green onion from two specific growers (vendors) tested positive for *Campylobacter*, *E. coli*, and *Listeria*, resulting in a higher risk of foodborne illnesses. Our detection of these pathogens, along with *Salmonella*, in fresh produce is particularly concerning as it poses potential harm to consumers, especially since most fresh produce receives minimal or no processing before consumption. This finding is especially timely given the recent multistate outbreaks of *Salmonella* [65] and *E. coli* [66] associated with fresh produce in the United States. Furthermore, due to the health risks related to these potential pathogen contaminations, there have been several food safety recalls involving fresh produce [67].

Additionally, a study has shown that, even if produce is washed in chlorinated water before consumption, foodborne pathogens in precut lettuce may not be eliminated and can continue to grow if stored at 10°C and above [68]. Therefore, the presence of foodborne pathogens on farmers′ market produce, which are not subject to temperature control, can be a serious food safety issue [20]. Besides, in‐depth investigation of the environment to figure out the factors contributing to the prevalence of foodborne pathogens in the commodities might help educate stakeholders including farmers and develop better mitigation strategies to control foodborne pathogens.

Among the 264 animal‐origin samples tested, *E. coli*, *Listeria* spp., and *Salmonella* spp. were detected in 76 (28.8%), 36 (13.6%), and 3 (1.1%) of the samples, respectively. *L. monocytogenes* was found in approximately 5.3% of the samples. Interestingly, none (0%) of the animal‐origin samples in our study tested positive for *Campylobacter*, despite its known association with poultry‐related outbreaks [56]. In contrast, *Campylobacter* was detected in 2.3% of fresh produce samples, suggesting the possibility of cross‐contamination occurring during or at some point between the farm and the store.

Among the 76 (28.8%) of the 264 animal‐origin samples that tested positive for *E. coli*, the majority of detections were found in chicken (77.5%, 31/40), followed by pork sausage (37.2%, 32/86), ground beef (12.0%, 6/50), and eggs (8.0%, 7/88). The overall prevalence (28.8%) in our study was comparable with the one found by Scheinberg et al. [48], which detected *E. coli* in 29.0% of samples overall, with 40% in beef and 18% in pork, from 50 beef and 50 pork samples collected from 25 farmers′ markets and 58 vendors in Pennsylvania. Although it is out of the scope of this study, Zhao et al. [69] reported high *E. coli* prevalence in ground beef (69%) and pork (44%) samples from supermarkets in several U.S. states, highlighting the widespread issue of unhygienic conditions in meat products in the United States. Given that *E. coli* presence in food products indicates possible fecal contamination during handling and processing, these findings are concerning, especially since pathogenic *E. coli* O157 can originate from the same contamination sources [52, 60–62].

Among the 36 (13.6%) of the 264 animal‐origin samples that tested positive for *Listeria* spp., *L. innocua*, *L. monocytogenes*, and *L. welshimeri* were isolated from 18, 14, and 4 samples, respectively. The most prevalent *Listeria* species in the samples were *L. monocytogenes*, accounting for 50% (18/36). The prevalence (13.6%) of *Listeria* spp. obtained from meat samples in our study was much higher to the study [48], which detected *Listeria* spp. in 4% overall, with 8% in beef and 0% in pork. In the latter study, among *Listeria* spp. isolates, 30% were identified as *L. monocytogenes*, whereas our study identified 38.9% as *L. monocytogenes*. Although the prevalence rate of *Listeria* spp. was comparable, differences between sample size and commodities evaluated could have led to the disparity in overall *Listeria* prevalence.

In assessing *Salmonella* presence, although the overall occurrence was low (1.1%, 3/264), the majority (66.7%, 2/3) of *Salmonella* was detected in ground beef samples. Concurrently, Larsen [70] reported a 2% prevalence of *Salmonella* in animal product samples from 44 certified Northern California farmers′ markets. However, Scheinberg [71] reported a significantly higher incidence, with 28% of whole chickens (*n* = 100) purchased from farmers′ markets in Pennsylvania being positive for *Salmonella* spp.

Overall, regardless, good handling practices are recommended due to the presence of potential pathogens in the studied fresh produce and animal‐origin samples. Differences among sample size, commodities, and mode of transportation used in those studies and our study could have led to the disparity in results. Local sanitary conditions during production, processing and retail, could also have contributed to the differences. Therefore, more information on contamination at different points in the production and supply chain of farmers and farmers′ market vendors is needed to interpret these differences.

Based on our previous research [9, 33] and the current study, which analyzed 332 and 740 food commodities, including fresh produce and animal‐origin samples from farmers′ markets in the same region of Virginia, we observed a decrease in the occurrences of *Campylobacter* (1.5%) and *Listeria* (7.3%) during the pandemic compared with prepandemic levels (5.1% for *Campylobacter* and 12.8% for *Listeria)*. However, *E. coli* occurrences increased during the pandemic (19.2%) compared with the prepandemic (17.5%). In contrast, the occurrence of *Salmonella* remained relatively consistent, with rates of 0.5% before and 0.8% during the pandemic. In addition, interestingly, the detection rates of *Campylobacter*, known as being prevalent in food animals [34], were lower in animal‐origin samples (1.5% prepandemic and 0% during the pandemic) than in fresh produce samples (8.7% prepandemic and 2.3% during the pandemic), possibly suggesting cross‐contamination at some point during or at the venue from farm to farmers′ markets.

Although not within the scope of the current study, earlier research [36, 37] on 387 food commodities from food desert markets in the same region before the pandemic revealed the prevalence of *Campylobacter* (6.9%), *E. coli* (15.7%), *Listeria* (4.8%), and *Salmonella* (2.4%). These findings suggest that *Campylobacter* (1.5%) and *Salmonella* (0.8%) rates in farmers′ markets during the pandemic were lower than the prepandemic rates in food desert corner markets. However, pandemic levels of *E. coli* (19.2%) and *Listeria* (7.3%) in farmers′ markets were higher than prepandemic levels of *E. coli* (15.7%) and *Listeria* (4.8%). As noted by Kim et al. [37], variations in sample commodities (such as fresh produce, meat products, and value‐added items), display methods (e.g., refrigeration versus open‐air), and transportation practices among farmers′ markets, supermarkets, and corner stores may explain the differences in results. Additionally, market sanitary conditions during production, processing, and retail could have contributed to these disparities. Therefore, further research into contamination at different stages of the production and supply chain related to various market sources is essential for a comprehensive understanding of these variations.

Although *Campylobacter*, *E. coli*, *Listeria*, and *Salmonella* were detected among the samples analyzed, no meaningful associations were observed between their occurrences (*r* < 0.1411; *p* > 0.0001). The weak correlations identified are consistent with earlier reports [9, 33], indicating that the presence of *E. coli* (commonly used as an indicator of fecal contamination, IMNRC [72]) does not reliably predict the occurrence of pathogenic microorganisms, and vice versa. These findings further suggest that relationships among indicator organisms and foodborne pathogens may vary depending on commodity type and environmental conditions.

### 3.5. AMR

**Figure 2:**
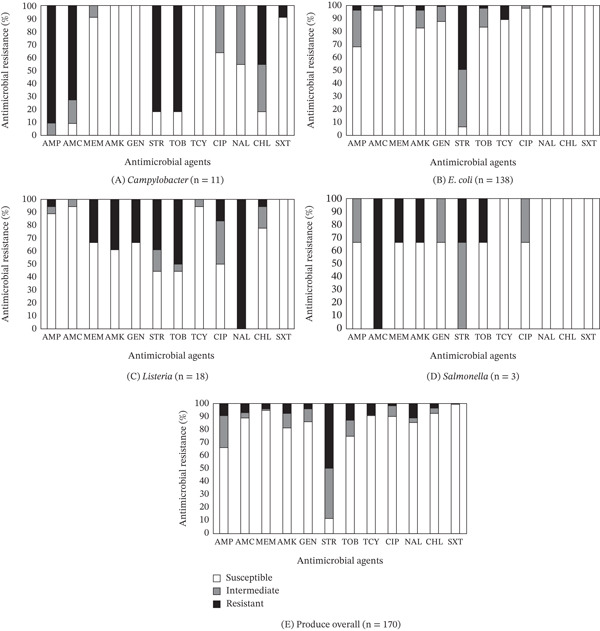
(A–E) Prevalence of resistance to 12 antimicrobial agents in a total of 170 bacterial isolates (A: *Campylobacter*, B: *E. coli*, C: *Listeria*, D: *Salmonella*, and E: overall) obtained from selected fresh produce samples procured from farmers′ markets in Central Virginia between August 2020 and December 2021.

The prevalence of *Campylobacter*, *E. coli*, *Listeria,* and *Salmonella* isolates resistant to the 12 antimicrobials tested is summarized in Table 4. The susceptible, intermediate, and resistant patterns of *Campylobacter* isolates obtained from fresh produce samples in relation to the antimicrobials tested in this study are presented in Figure 2A. Of the 11 *Campylobacter* isolates, resistance to AMP was the most common in 10 (90.9%) isolates, followed by STR (9 isolates, 81.8%), TOB (9 isolates, 81.8%), AMC (8 isolates, 72.7%), CHL (5 isolates, 45.5%), and SXT (1 isolate, 9.1%). All of the *Campylobacter* isolates tested were resistant to at least two antimicrobial agents (Table 4). A study by Kim et al. [9] on AMR in 12 *Campylobacter* isolates from fresh produce samples collected at farmers′ markets in the same region prior to the pandemic also revealed a similar resistance pattern, with 100% resistance to AMP and 91.7% to AMC. In the present study, three (27.3%) isolates from tomato and squash displayed nonsusceptibility to six antimicrobials, whereas one isolate from squash showed nonsusceptibility to eight antimicrobials. Additionally, MDR was identified in nine (81.8%) *Campylobacter* isolates in the current study, compared with 11 (91.7%) isolates obtained before the pandemic. Although the current study found that AMK, GEN, and TCY exhibited 100% susceptibility against all *Campylobacter* isolates obtained during the pandemic, none of the antimicrobials tested were effective against all *Campylobacter* isolates collected prior to the pandemic.

Among the 138 *E. coli* isolates obtained from fresh produce samples, resistance to STR was the most common in 68 (47.8%) isolates, followed by TCY (15 isolates, 10.9%), AMP (5 isolates, 3.6%), and AMK (5 isolates, 3.6%) (Figure 2B). Only 41 (29.7%) isolates were susceptible to all tested antimicrobials, indicating that 70.3% of *E. coli* isolates were nonsusceptible to at least one antimicrobial (Table 4). Two (1.4%) *E. coli* isolates, one from arugula and one from lettuce, showed MDR to the antimicrobials tested in this study. In specific, one isolate showing the highest level of nonsusceptibility was obtained from an arugula sample and displayed nonsusceptibility to seven antimicrobials. Interestingly, although this study found that CHL and SXT exhibited 100% susceptibility against all *E. coli* isolates obtained during the pandemic (Figure 2B), none of the antimicrobials tested were effective against all *E. coli* isolates obtained from farmers′ markets in the same region prior to the pandemic [9]. In their study, among the 23 *E. coli* isolates tested, resistance to AMP was the most common, observed in 11 (47.8%) isolates, followed by resistance to STR in 8 (34.8%) isolates, AMC in 6 (26.1%) isolates, TCY in 2 (8.7%) isolates, SXT in 2 (8.7%) isolates, and NAL in 1 (4.3%) isolates. Only three (13.0%) isolates were susceptible to all tested antimicrobials, indicating that 87.0% of *E. coli* isolates were nonsusceptible to at least one antimicrobial. Additionally, four (17.4%) *E. coli* isolates from bell pepper and sweet potato exhibited MDR to the antimicrobials tested.

The prevalence of resistance to antimicrobials in 18 *Listeria* isolates obtained from fresh produce samples tested in this study revealed that NAL resistance was the most common (18 isolates, 100%), followed by resistance to TOB (9 isolates, 50.0%), AMK (7 isolates, 38.9%), STR (7 isolates, 38.9%), MEM (6 isolates, 33.3%), and GEN (6 isolates, 33.3%) (Figure 2C). One isolate each of *L. welshimeri* and *L. monocytogenes* from squash exhibited resistance to eight and seven antimicrobials, respectively. Four isolates‐including two *L. innocua*, one *L. monocytogenes*, and one *L. welshimeri*‐from green beans, kale, and squash showed resistance to six antimicrobials. Additionally, eight isolates demonstrated MDR. These findings align with our previous research on fresh produce samples collected from farmers′ markets before the pandemic [9]. Both studies consistently revealed that CIP was the only antimicrobial that exhibited 100% susceptibility against all *Listeria* isolates. In the earlier study, among the 11 *Listeria* isolates, resistance to NAL was most common, detected in eight isolates (72.7%), followed by resistance to AMP in seven isolates (63.6%), GEN in six isolates (54.5%), STR in six isolates (54.5%), and TCY in six isolates (54.5%). Both studies also revealed that all *Listeria* isolates tested were resistant to at least one antimicrobial agent.

Among the three *Salmonella* isolates, resistance to AMC was the most prevalent in all (100%) isolates. In contrast, resistance to MEM, AMK, STR, and TOB was each detected in a single isolate (33.3%) (Figure 2D). Although none of the isolates were susceptible to all tested antimicrobials (Table 4), one isolate obtained from a kale sample displayed the highest resistance and nonsusceptibility to five and seven antimicrobials, respectively. The current study also revealed that TCY, NAL, CHL, and SXT exhibited 100% susceptibility against all *Salmonella* isolates obtained (Figure 2D).

The present survey on fresh produce samples collected at farmers′ markets during the pandemic revealed that the prevalence of MDR to 12 antimicrobials tested in the study was the highest in *Campylobacter* (81.8%), followed by *Listeria* (44.4%), *Salmonella* (33.3%), and *E. coli* (1.4%) (Table 4). Among all the antimicrobials tested against bacteria isolated from fresh produce samples in this study, AMP, STR, NAL, and AMC exhibited the highest resistance rates, with *Campylobacter* showing 90% resistance, *E. coli* 50%, *Listeria* 100%, and *Salmonella* 100%, respectively (Figure 2A–D). These findings align with similar resistance patterns observed in bacteria isolated from fresh produce samples collected from farmers′ markets prior to the pandemic [9]. The most effective antimicrobials identified in this study were AMK, GEN, and TCY for *Campylobacter*; CHL and SXT for *E. coli*; SXT for *Listeria*; and TCY, NAL, CHL, and SXT for *Salmonella*. Across all 170 bacterial species tested, STR identified to be the least effective, with 90% nonsusceptibility, whereas SXT was the most effective, showing 99.5% susceptibility (Figure 2E).

**Figure 3:**
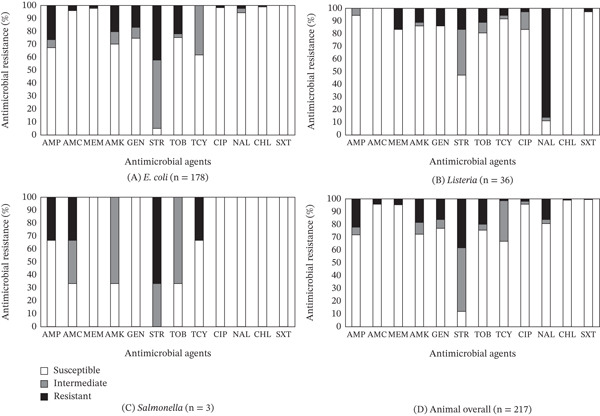
(A–D) Prevalence of resistance to 12 antimicrobial agents in a total of 217 bacterial isolates (A: *E. coli*, B: *Listeria*, C: *Salmonella*, and D: overall) obtained from selected animal‐origin samples procured from farmers′ markets in Central Virginia between August 2020 and December 2021.

The susceptibility, intermediate, and resistance profiles of *E. coli*, *Listeria*, and *Salmonella* isolates from animal‐origin samples, in response to the antimicrobials tested in this study, are illustrated in Figure 3A–C. Of the 178 *E. coli* isolates, resistance to STR was the most common in 75 (42.1%) isolates, followed by AMP (26.4%), TOB (21.9%), AMK (20.2%), and GEN (16.9%) (Figure 3A). Among the *E. coli* isolates tested, only 34 (19.1%) isolates were susceptible to all antimicrobials, indicating that 80.9% exhibited nonsusceptibility to at least one antimicrobial (Table 4). Five isolates (2.8%), all from pork sausage, showed MDR, with one particular isolate demonstrating the highest level of nonsusceptibility to nine antimicrobials. The findings of this study revealed that *E. coli* isolates collected during the pandemic were entirely (100%) susceptible to SXT (Figure 3A). This contrasts with the findings of a prepandemic study by Kim et al. [33] on 118 *E. coli* isolates from value‐added meat products at farmers′ markets in the same region. That study revealed different resistance patterns, with none of the antimicrobials tested being fully effective. Resistance to TCY was most common (29.7%), followed by AMP (28.8%), STR (22.0%), and AMC (14.4%). Similarly, another study by Kim et al. [32] on *E. coli* isolated from farm animals and meat‐origin food samples in the eastern United States found a 62.1% resistance rate to TCY. Notably, Kim et al. [33] study also reported an isolate from pork sausage with the highest nonsusceptibility to 10 antimicrobials. Our findings (80.9% nonsusceptibility to at least one antimicrobial) are consistent with previous studies, where 82.2% [33] and 98.5% [32] of *E. coli* isolates were found to be nonsusceptible to at least one antimicrobial. MDR was identified in 19 (16.1%) *E. coli* isolates collected before the pandemic.

The prevalence of resistance to antimicrobials in 36 *Listeria* isolates obtained from animal‐origin samples tested in this study revealed that NAL resistance was the most common (31 isolates, 86.1%), followed by resistance to MEM (6 isolates, 16.7%), STR (6 isolates, 16.7%), and GEN (5 isolates, 13.9%) (Figure 3B). One *L. innocua* isolate from pork sausage and one *L. welshimeri* isolate from ground beef exhibited resistance to seven and six antimicrobials, respectively. Additionally, another *L. innocua* isolate from pork sausage and *L. welshimeri* isolate from ground beef exhibited resistance to six antimicrobials and nonsusceptibility to seven antimicrobials, respectively. Furthermore, four isolates (11.1%) demonstrated MDR. These findings are comparable to our previous study on value‐added commodities collected from farmers′ markets before the pandemic [33]. The previous study on 34 *Listeria* isolates revealed that MDR was identified in 20 isolates (58.8%) whereas NAL resistance was the most common in 27 isolates (79.4%), followed by resistance to MEM (15 isolates, 44.1%), AMK (13 isolates, 38.2%), and GEN (13 isolates, 38.2%). Additionally, approximately 97% of the *Listeria* isolates tested in the current study and Kim et al. [33] study were nonsusceptible to at least one antimicrobial. In contrast to our current findings, where AMC and CHL exhibited 100% susceptibility (Figure 3B), Kim et al. [33] study found that although TCY showed 64.7% susceptibility, none of the antimicrobials tested demonstrated 100% susceptibility against *Listeria* isolates obtained before the pandemic.

Of the three *Salmonella* isolates obtained from animal‐origin samples, resistance to STR was the most prevalent, observed in two isolates (66.7%), whereas resistance to AMP, AMC, and TCY was each found in one isolate (33.3%) (Figure 3C). Although two isolates obtained from ground beef were nonsusceptible to five antimicrobials, only one isolate obtained from the ground beef sample displayed MDR. The current study also revealed that MEM, GEN, CIP, NAL, CHL, and SXT exhibited 100% susceptibility against all *Salmonella* isolates obtained (Figure 3C). These findings are comparable with our previous study on value‐added commodities collected from farmers′ markets before the pandemic [33]. Kim et al. [33] study on two *Salmonella* isolates obtained revealed that both isolates were identified to be MDR while resistance to AMP, AMC, STR, and TCY was the most common at 100%, followed by TOB at 50.0%. The isolates were susceptible to MEM, AMK, CIP, and SXT. Particularly, for Kim et al. [33] study, one isolate obtained from fresh egg was nonsusceptible to eight antimicrobials with resistance to five antimicrobials. In addition, a previous study [32] on the prevalence of AMR in 121 *Salmonella* isolates obtained from farm animals, wildlife, and food samples in the eastern United States revealed their susceptibility to two antimicrobials (MEM and AMK) only. Drawing from the findings of our studies, along with those of others [73–76], the susceptibility of *Salmonella* to MEM and AMK demonstrates the effectiveness of these antimicrobials in treating *Salmonella* infections in both veterinary and human medicine. Additional studies [32, 77, 78] that examined shell eggs from poultry farms, farmers′ markets, and commercial sources similarly found 100% susceptibility of *Salmonella* to MEM and AMK. Regarding the differences in AMR among studies, Doyle et al. [79] suggested that variability in the prevalence and diversity of AMR across countries and regions may be attributed to significant differences in antimicrobial use and practices.

The present survey on animal‐origin samples revealed that the prevalence of MDR to 12 antimicrobials tested in the study was the highest in *Salmonella* (33.3%), followed by *Listeria* (11.1%) and *E. coli* (2.8%) (Table 4). Among all the antimicrobials tested, STR, NAL, and STR exhibited the highest resistance rates, with *E. coli* showing 42.1% resistance, *Listeria* 86.1%, and *Salmonella* 66.7%, respectively (Figure 3)A–C. These findings align with similar resistance patterns observed in bacteria isolated from value‐added commodities collected from farmers′ markets prior to the pandemic [33]. The most effective antimicrobials identified in this study were AMK for *E. coli*; AMC and CHL for *Listeria*; and MEM, GEN, CIP, NAL, CHL, and SXT for *Salmonella*. Across all 217 bacterial species tested, STR identified to be the least effective, with 88.0% nonsusceptibility, whereas SXT was the most effective, showing 99.5% susceptibility (Figure 3D).

Overall, as no *Campylobacter* was detected in samples of animal origin in the present survey, a comparison of AMR prevalence between isolates from fresh produce and those from animal‐derived samples could not be conducted. However, *E. coli* isolates obtained from fresh produce samples exhibited lower MDR (1.4%) than those (2.8%) obtained from animal‐origin samples, whereas *Listeria* isolates obtained from fresh produce samples exhibited higher MDR (44.4%) than those (11.1%) obtained from animal‐origin samples (Table 4). MDR prevalence of *Salmonella* isolates obtained from fresh produce and animal‐origin samples was the same at 33.3%. Additionally, 47.8% of *E. coli* isolates from fresh produce and 42.1% from animal‐origin samples exhibited the highest resistance to STR, whereas 100% of *Listeria* isolates from fresh produce and 86.1% from animal‐origin samples displayed the highest resistance to NAL. In contrary, the highest AMR noted in *Salmonella* isolates from fresh produce samples was 100% against AMC, whereas the highest AMR in isolates from animal‐origin samples was 66.7% against STR. Distinct patterns emerged regarding the most effective antimicrobials for *E. coli* isolates, with CHL and SXT being effective against bacteria from fresh produce (Figure 2B), whereas SXT was effective against those from animal‐origin samples (Figure 3A). For *Listeria*, SXT was effective against isolates from fresh produce (Figure 2C), whereas AMC and CHL were effective against isolates from animal‐origin samples (Figure 3B). However, *Salmonella* isolates from both sample types were susceptible to NAL, CHL, and SXT (Figures 2D and 3C).

**Figure 4:**
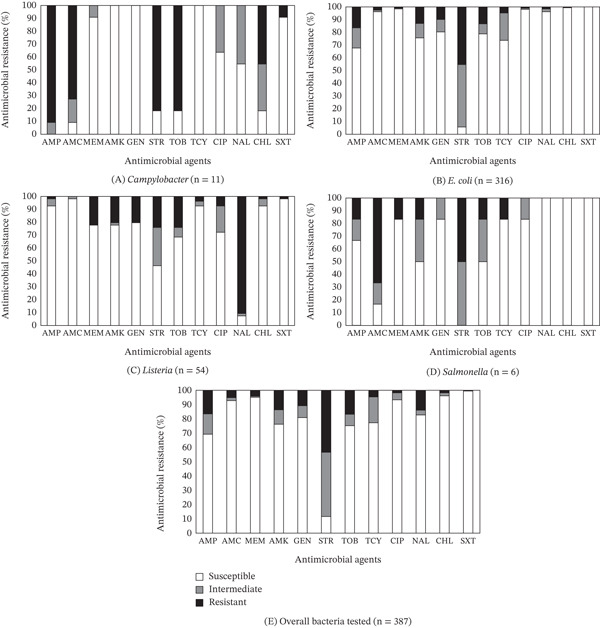
(A–E) Overall prevalence of resistance to 12 antimicrobial agents in each type of bacteria (A: *Campylobacter*, B: *E. coli*, C: *Listeria*, D: *Salmonella*, and E: overall) obtained from both fresh produce and animal‐origin samples.

Regardless of sample types, the present survey on all samples collected at farmers′ markets during the pandemic revealed that the prevalence of MDR was the highest in *Campylobacter* (81.8%), followed by *Salmonella* (33.3%), *Listeria* (22.2%), and *E. coli* (2.2%) (Table 4). Among all the antimicrobials tested, AMP, STR, NAL, and STR exhibited the highest nonsusceptibility rates, with *Campylobacter* showing 100%, *E. coli* 94.3%, *Listeria* 92.6%, and *Salmonella* 100%, respectively (Figure 4A–D). The most effective antimicrobials identified in this study were AMK, GEN, and TCY for *Campylobacter*, SXT for *E. coli*, AMC for *Listeria*, and NAL, CHL, and SXT for *Salmonella*. Across all 387 bacterial species tested, STR identified to be the least effective, with 88.4% nonsusceptibility, whereas SXT was the most effective, showing 99.5% susceptibility (Figure 4E).

For the isolates obtained before the pandemic, the prevalence of MDR was the highest in *Salmonella* (100%), followed by *Campylobacter* (95.9%), *Listeria* (56.7%), and *E. coli* (16.8%) [9, 33]. Of all the antimicrobials tested on bacteria isolated from the samples, AMP, AMP, and NAL showed the highest rates of nonsusceptibility, with *Campylobacter* at 100%, *E. coli* at 62.4%, and *Listeria* at 86.7%, respectively. *Salmonella* demonstrated 100% nonsusceptibility to multiple antimicrobials, including AMP, AMC, STR, and TCY. Although none of the tested antimicrobials were effective against *Campylobacter* beyond 33.3%, the previous study identified MEM as the most effective antimicrobial for *E. coli* (99.3% susceptibility) and SXT for *Listeria* (75.6% susceptibility). Meanwhile, *Salmonella* was found to be 100% susceptible to MEM, AMK, CIP, and SXT. Across all 203 bacterial species tested, AMP identified to be the least effective, with 61.1% nonsusceptibility, whereas SXT was the most effective, showing 88.1% susceptibility. In this regard, Kim et al. [32] observed that bacterial isolates can develop different resistance levels depending on the environmental conditions they encounter and their genetic background. Furthermore, Karumathil et al. [80] reported the prevalence of MDR in opportunistic human pathogens (*Acinetobacter baumannii, Burkholderia cepacia, Stenotrophomonas maltophilia*, and *Pseudomonas luteola*) associated with farmers′ market‐acquired food products, highlighting the potential health risk in consumers, especially those with a compromised immune system.

Therefore, the information presented underscores the need to raise awareness among stakeholders, including food producers and consumers, about the cautious and responsible use of antimicrobials from a One Health perspective to help prevent the future emergence of AMR bacteria in foodborne illnesses. The results from this study and our previous research [9, 32, 33] on fresh produce and value‐added products—though limited to a specific geographical area—generally reflect a consistent pattern between the prevalence of resistance to certain antimicrobial categories and their usage levels in the United States between 2000 and 2010 [81].

Additionally, several limitations should be acknowledged when interpreting the findings of this study. Sampling was restricted to farmers′ markets within a 50‐km radius of Virginia State University in Central Virginia, which may limit the broader applicability of the results to farmers′ markets operating in other regions with differing climatic conditions, regulatory frameworks, and production practices. Although multiple food commodity types were included, product availability varied by market and season, leading to uneven sample sizes across commodities and vendors that may have influenced observed pathogen prevalence and AMR patterns. The relatively small number of isolates recovered for certain pathogens, particularly *Campylobacter* and *Salmonella*, further constrained the ability to perform robust comparative analyses across commodity types, markets, and time periods. Moreover, although comparisons were made between prepandemic and pandemic datasets, the study did not directly measure vendor or consumer compliance with PPE use, sanitation practices, or food handling behaviors, nor did it evaluate environmental or on‐farm sources of contamination. Despite these limitations, the study provides valuable baseline data on microbial occurrence and AMR profiles of foods sold at farmers′ markets during the COVID‐19 pandemic and identifies important directions for future research and surveillance.

## 4. Conclusions

The research reveals that although the overall prevalence of major foodborne pathogens was relatively low, *E. coli*, *Listeria*, *Campylobacter*, and *Salmonella* were all detected. Compared with prepandemic data, the occurrence of *Campylobacter* and *Listeria* declined, whereas *E. coli* and *Salmonella* showed modest increases, suggesting shifts in microbial contamination patterns during the pandemic period. Correlation analysis confirmed a weak association between *E. coli* and other foodborne pathogens, reinforcing evidence that although *E. coli* serves as an indicator of fecal contamination and general hygiene, it is not a reliable predictor of the presence of specific pathogenic bacteria. AMR analysis revealed *Campylobacter* as the most prevalent MDR organism, consistent with prepandemic findings, although resistance patterns differed between periods. STR was the least effective antimicrobial during the pandemic, whereas SXT remained the most effective, highlighting evolving resistance trends. Collectively, the detection of foodborne pathogens despite the implementation of PPE and pandemic‐related safety measures underscores the persistent public health risks associated with farmers′ market products. These findings emphasize the continued need for robust agricultural practices, proper handling, and targeted food safety interventions. Moreover, the study highlights the importance of ongoing surveillance, education, and training to raise awareness among farmers, vendors, consumers, and policymakers and to support the development of effective food safety programs aimed at reducing foodborne illness risks.

## Author Contributions

Chyer Kim, Eunice Ndegwa, and Theresa Nartea conceived the original idea. Chyer Kim performed statistical analysis, interpreted data, and wrote the manuscript. Abeer Abujamous, Daria Clinkscales, and Allissa Riley carried out the experiment. Theresa Nartea surveyed farmers′ markets in Virginia and edited manuscript. Salina Parveen, Junglim Lee, Eunice Ndegwa, and Theresa Nartea interpreted data and edited manuscript. Chyer Kim generated a map of Virginia showing locations of farmers′ markets using a geographic information system software (ArcGIS).

## Funding

This study was supported by U.S. Department of Agriculture (10.13039/100000199, 2020‐38821‐31082).

## Disclosure

The authors declare that this study was conducted primarily for academic research purposes without any conflict of interest. Any mention of trade names or commercial products is solely for informational purposes and does not imply endorsement or recommendation by Virginia State University. The study examines the occurrence and AMR profiles of selected foodborne pathogens in randomly chosen food commodities from various farmers′ markets in Central Virginia, United States, during the COVID‐19 pandemic. Although the limited number of bacterial isolates and the restricted availability of certain commodities from different vendors may prevent the results from fully representing all *Campylobacter*, *E. coli*, *Listeria*, *Salmonella*, and food commodities in the region, the findings are valuable for understanding the broader occurrence and AMR of these pathogens in farmers′ market products. All authors discussed the results and contributed to the final manuscript.

## Conflicts of Interest

The authors declare no conflicts of interest.

## Data Availability

The data that support the findings of this study are available from the corresponding author upon reasonable request.
